# Unmet Cardiac Clinical Needs in Adult Mucopolysaccharidoses

**DOI:** 10.3389/fcvm.2022.907175

**Published:** 2022-06-10

**Authors:** Karolina M. Stepien, Elizabeth A. Braunlin

**Affiliations:** ^1^Inherited Metabolic Diseases Department, Salford Royal NHS Foundation Trust, Salford, United Kingdom; ^2^Department of Pediatrics, University of Minneapolis, Minneapolis, MN, United States

**Keywords:** adult mucopolysaccharidoses, cardiac, unmet needs, therapies, transition

## Abstract

The Mucopolysaccharidoses (MPSs) are a group of heterogenous disorders with complex multisystemic presentations. Although Haematopoietic Cell Transplantation (HCT) and Enzyme Replacement Therapy (ERT) have extended the lifespan of individuals affected with MPS well into adulthood, reversal of pre-existing cardiac, skeletal and neurocognitive deficits does not occur, so there are no truly curative treatments available to these patients at present. The medical and surgical management of cardiovascular problems in adults with MPS is complicated by these pre-existing comorbidities, requiring the involvement of multidisciplinary and multispecialty perioperative teams. This review sets out to describe the unmet cardiac needs in adults with MPS disorders including the lack of effective treatments, monitoring guidelines, and the challenges regarding expertise and training, and psychosocial support.

## Introduction

Rare diseases, defined as conditions occurring less frequently than 1:2,000 (1), pose a significant challenge to healthcare systems worldwide. More than 6,000 rare diseases are currently known; in excess of 70% of these are thought to have a genetic component and it is estimated that more than 300,000,000 individuals are affected worldwide with some type of rare disease (2). Delayed diagnosis and misdiagnosis are common, resulting in incorrect treatment and poor clinical outcomes (3, 4). Many genetically determined rare diseases exist as isolated cardiac conditions (i.e., left ventricular non-compaction cardiomyopathy, long QT syndrome) or as part of a systemic phenotype [i.e., Marfan syndrome, Fabry disease, the mucopolysaccharidoses (MPSs)].

The MPSs (types I, II, III, IV, VI, VII, and X) are caused by deficiencies of type-specific lysosomal enzymes leading to excessive deposition of glycosaminoglycans (GAGs) in cardiovascular, skeletal and central nervous system tissues that ultimately affects their function (5, 6). Severe forms of MPS are often identified in infancy or childhood while attenuated forms may not be found until adolescence or adulthood, if at all. Depending upon the type of MPS and the age as well as the clinical picture at presentation, hematopoietic cell transplantation (HCT) and enzyme replacement therapy (ERT) are current treatment options. Before these options were available, the severest forms of MPS were considered lethal diseases of childhood and the attenuated forms were associated with shortened lifespans. Although HCT and ERT have extended the lifespan of individuals affected with MPS well into adulthood, reversal of pre-existing cardiac, skeletal and neurocognitive deficits does not occur so there is no truly curative treatment available at present. The medical and surgical management of cardiovascular problems in adults with MPS is complicated by these pre-existing comorbidities. For adults–both those treated as young children by HCT as well as those identified later in adulthood and under current ERT-the MPSs have become chronically debilitating conditions of adulthood, which place a significant burden on the affected individual, their families, and society/healthcare systems. This mini-review outlines the unmet cardiac needs in adults with MPS disorders, with the focus on managing their symptoms, psychosocial support and long-term monitoring.

## Global Unmet Needs

### Access to Therapies

It is estimated that approximately 95% of rare diseases have no treatment options (7). Even if a therapy has been made available, developing countries often cannot afford it or have limited access to it. Some countries still lack orphan drug policies (8, 9) which make it difficult for patients to access these treatments. Patient organizations can advocate for achievable and realistic policy changes based on experiences of countries/areas of the same economic area (8). The lack of treatment options and inability to access drugs that are available in other territories but not licensed in some countries, such as Australia, is a cause for frustration among clinicians (9). On a global scale, disparities subsequently developed both in the establishment and/or implementation of governmental rare disease policies and in equitable access to therapies (8), most usually based upon underlying wealth of individual countries.

### Efficacy of Therapies

Long term efficacy of current treatments is often unavailable. Natural history studies of MPS disorders provide insight into the long-term effect of various therapies. Such longitudinal data can help identify unmet needs and encourage further research. There is however an increasing need for more therapies which affect the central nervous system (CNS) and arrest neurocognitive decline (10, 11).

For MPS, currently available therapies such as HCT have been shown to be effective, in the short term, in slowing disease progression in certain types of MPS (12–18). In other types of MPS (I, II, IV and VI), ERT as a supportive treatment has shown benefits regarding GAG reduction and increased endurance during the 6-min walk test (12–18).

Clinical outcomes of future therapies could focus more on patient reported outcomes (PROs) and health related quality of life (HrQoL). Consequently, the emotional, psychological and economical impact of a rare disease on patients and their families should have a greater significance attached; studies have demonstrated the benefits of a more holistic approach (19).

The passage of the Rare Disease Act (1983) in the United States and the European Union (EU) Regulation 141/2000 (1999) resulted in a significant increase in new drug approvals (primarily ERTs) for MPS that extended survival but also brought with them a whole new collection of issues. Patients and their families justifiably advocated for approval of these promising therapies with governmental regulatory agencies. This, in turn, led to approval of treatments whose outcomes were often based upon clinical trials of limited duration with no established endpoints for assessing lack of efficacy (20).

### Guidelines

More importantly, since the introduction of the first ERTs for MPS, there is both a plethora of expert opinion and a dearth of data-driven evidence for the long-term efficacy of current therapies. Multiple treatment guidelines have been developed for all types of MPS, most financed, with rare exception, at arms' length by the pharmaceutical companies who developed the same treatments (20). These guidelines can differ from evidence-based reviews performed independently (20, 21) and represent the most significant unmet need for MPS. The scarcity of experts in specific rare disease sub-specialties (i.e., cardiology) can also lead to bias in guideline development.

Fundamental to obtaining reliable outcomes data for current and emerging therapies for rare diseases like MPS are the need for uniform rare disease medical codes (22, 23), global registries that capture the new natural histories of MPS, data-driven unbiased guidelines, and training programs for next-generation of medical professionals to care for these individuals. The coordination of care via centers of expertise is key to integrated programmes and strategies for rare diseases (24, 25). As initiatives to improve coordination vary among countries (24, 25) the role of patient organizations is essential in the development of uniform programmes to address the needs of the rare disease community (24).

Apart from ERT and HCT for MPS, novel treatments including substrate reduction therapy, pharmacological chaperone therapy, and gene therapy have become available (20). Therefore, there is an increasing need for optimizing and standardizing guidelines for each MPS type, with a focus on therapeutic efficacy, adverse effects, age, disease stage, prognosis, feasibility and availability of access to treatment, and cost-effectiveness (20).

The increased survival of MPS patients has created a number of new issues such as the development of age-related complications and the lack of data on the natural long-term course of the disease (26). The lack of standardized programs or specific pre-operative and more general treatment protocols/guidelines shared among adult metabolic centres is another important aspect that needs to be addressed by institutions and policy makers to ensure the best possible care for adult MPS patients being considered for surgical interventions such as valve replacement surgery.

The EU has led the way in the enactment of well-integrated rare disease policy, the establishment of rare disease diagnostic codes, useable patient registries, a central repository for information on drugs for rare diseases, and a resource-rich web-based portal open to all (27). The challenge is for global cooperation to advance the understanding of MPS for the betterment of patients' lives.

## National Unmet Needs

**Figure 1:**
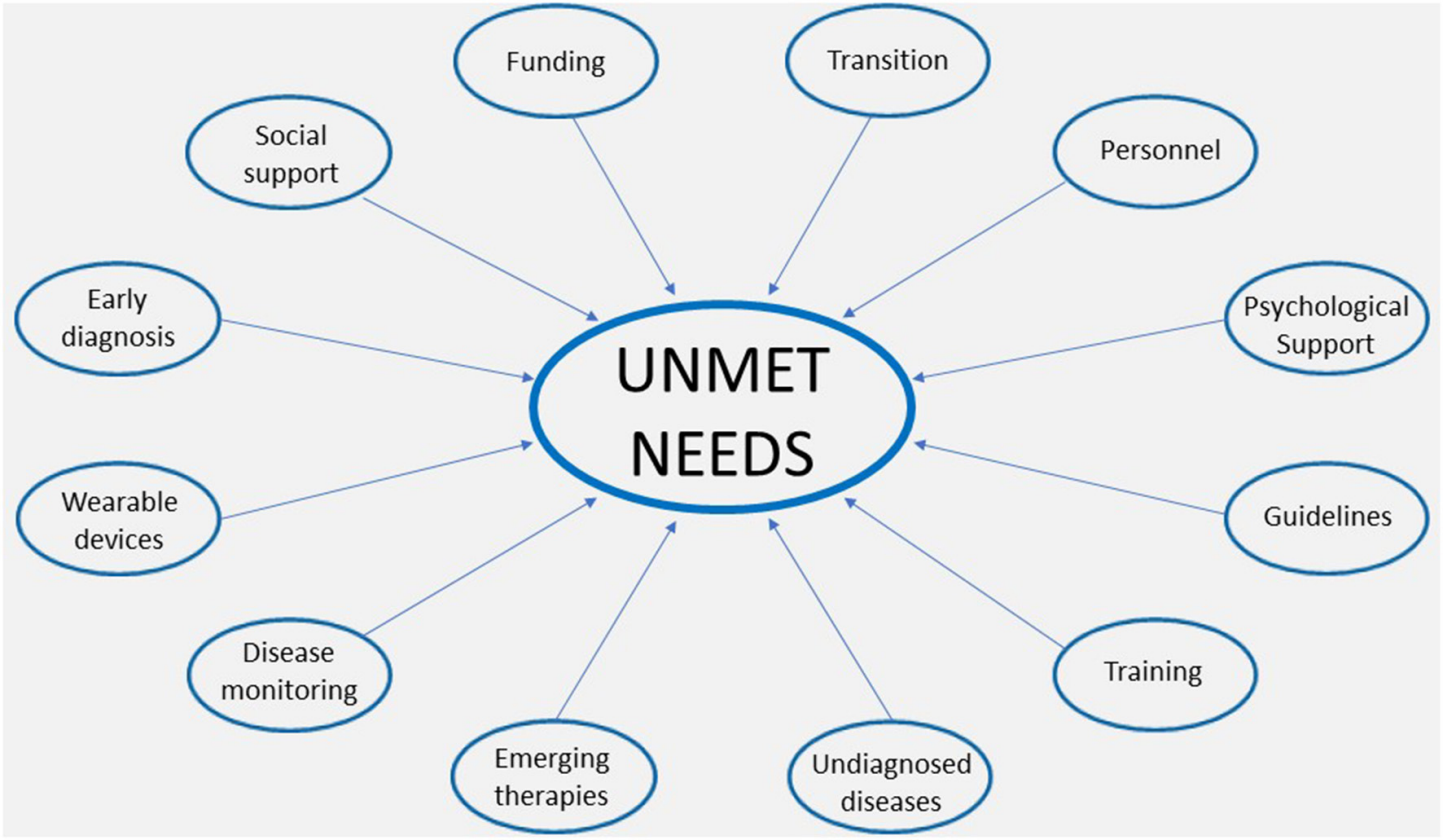
Unmet needs in adult patients with Mucopolysaccharidosis.

The unmet needs at a national level can be divided into doctors' unmet educational needs and patients' unmet patients' needs (Figure 1), as described below.

### Training

The need for formal training opportunities in adults and courses on inherited metabolic diseases (IMD) has been identified as an educational need in several surveys in the last few years (9, 28). Lack of specialty training has been previously recognized as an educational gap in training on IMDs among hospital clinicians (9, 26, 29, 30) as well as primary care doctors (28). For example, there is no curriculum for rare cardiovascular diseases in the US Adult Congenital Cardiology subspecialty fellowship manual (31), which limits the training opportunities of the cardiac aspect of MPS. In the UK, the entry into a 5-year pediatric cardiology training is possible either following completion of the Core Pediatric Level 1 curriculum and having obtained MRCPCH qualification or following completion of 2 years of the Internal Medicine stage 1 curriculum having obtained MRCP. Internal Medicine Trainees will however have to have demonstrated additional core pediatric and neonatal capabilities prior to commencing the Pediatric Cardiology curriculum (32). Additional training in congenital heart diseases can be obtained during fellowship in a specialist centers in the country or abroad.

The complexity of MPS conditions require highly specialized knowledge and experience, and although the IMD specialty offers training opportunities, it does not create novel interest among junior doctors (26). Limited MPS awareness among patients, families and physicians is the major reason for the difficulty in diagnosis of these conditions. It is important to establish a group of experts in each country with the aim of improving specialist training and communication.

### Personnel

Shortage of adult metabolic physicians with an expertise in the management of adult MPS is another recognized need (26, 29). Specialists in either respiratory, cardiac or orthopedic aspects of adult MPS are part of the infrastructure in adult metabolic centre, which is a key factor for a fully functioning pediatric to adult transition service. As the life span of adult MPS patients improves, the overall prevalence of adult patients has increased. Among them, the proportion of patients with more complex disorders and with neurocognitive dysfunction has also increased, requiring complex specialist care.

### Transition of Care

Earlier diagnosis and therapeutic developments have improved the life span of MPS patients over the last decades, with the majority of patients now surviving beyond the age of 20 years (33, 34). This has posed a new challenge: as they grow up, the care of IMD patients' needs to be transferred from metabolic pediatricians to adult metabolic physicians specialized in treating adults (26, 35). The transition process is critical to ensure that adolescent patients with IMDs obtain the best quality of life possible as adults. Young adults with MPS and families need to become empowered and take responsibility for their health (26).

Unfortunately, transition service has not been well developed in many countries and therefore patients remain under the pediatric team lifelong. In the US, Brazil or most European countries, genetic and metabolic disorders remain within the pediatric realm and as a result few adult physicians are aware of rare diseases. In the USA, formal transition of metabolic patients to adult services is very infrequent due to health system organization and lack of accepting providers. Training in clinical genetics does not include competencies of participating in care for adults with IMDs, including adult MPSs. The transition policies and tools are available, but do not specify specific clinical needs for individual disorders such as complex MPS disorders (36, 37).

### Diagnostic Odyssey

More complex disorders and with neurocognitive disabilities, and a greater number of IMDs presenting in adulthood are a result of the new genomic era. The 100,000 genome project was launched as a pilot study of genome sequencing in a national health care system in the UK. It showed an increase in diagnostic yield across a range of rare diseases (38). It has proven that whole genome sequencing (WGS) can uncover new diagnoses for people across the broadest range of rare diseases investigated to date and could deliver enormous benefits across national health services (38). The NIH Undiagnosed Disease Network (39) aims to improve the level of diagnosis and care for patients with undiagnosed diseases, to facilitate research into the etiology of undiagnosed conditions by collecting and sharing standardized, high-quality clinical and laboratory data including genotyping, phenotyping, and exposure to environmental factors, and to create an integrated and collaborative community across multiple clinical sites (39).

The diagnosis of MPS may be particularly difficult in attenuated forms. The red flags signs for early diagnosis of attenuated MPS disorders include heart valve disease as well as corneal clouding, non-inflammatory joint stiffness, juvenile carpal tunnel syndrome, hernias and hepatomegaly (40). An unexplained mitral or aortic valve disease and/or left ventricular hypertrophy, abnormal diastolic function, coronary artery disease, pulmonary hypertension and arrhythmia (41) should make any cardiology specialist consider cardiac gene panels for rare diseases. It may help make a diagnosis of MPS in the absence of other classical features.

The expanded use of next generation sequencing both widens the phenotypic spectrum within known diseases and reveals new IMDs. Another example is the Genetics of Learning Disability (GOLD) study established in Cambridge in 2001 which aims to identify mutations in novel genes on the X chromosome that are associated with intellectual disability and to better understand the mechanisms by which intellectual disability occurs. This study proved to be helpful for example in diagnosing a case of adult-onset MPS IIIA in a patient with only mild learning difficulties (42).

### Disease Monitoring

Several biomarkers have been used in lysosomal storage diseases, with urine GAGs as a diagnostic and monitoring test for MPS disorders. Adult MPS patients develop cardiovascular complications despite available therapies and often require heart valve surgery, in rare cases they develop heart failure (41). Progressively increasing B-Natriuretic Peptide (BNP) titer in asymptomatic valvular heart disease patients points to advancing valve disease. BNP adds important incremental prognostic information that is useful for valve patient management and for optimal timing of surgery in particular. Its utility specifically in MPS is yet to be formally clarified and defined (43). Progressive cardiac disease in adult MPS patients would benefit from sensitive and specific biomarkers.

Echocardiography and electrocardiography are key diagnostic techniques for the evaluation of valves, ventricular dimensions and function, and regular monitoring (41). Echocardiogram however is not sufficient to monitor adult MPS patients with cardiac valve insufficiency long-term. Lack of international recommendations that would ensure reproducibility of the echocardiographical measurements remains a challenge. In addition, echocardiography provides only limited information in the assessment of coronary artery anatomy and pathology (41).

Wearable technology (e.g., a FitBit™ device or Kardia Alive Cor™) allows continuous monitoring and potential early identification of serious or life threatening bradyarrhythmias among patients with all MPS types (44–47). Currently these are only used as per individual patient choice or as part of research. In future, the technology could be used for cardiovascular, sleep, saturation and physical activity monitoring.

Increasing digital connectivity by using remote consultations (telemedicine) to discuss MPS related health problems has become more frequently used in the last year of the Covid-19 pandemic. It allows patients and physicians to connect with the multidisciplinary team (MDT), allowing access to experts based in different hospitals. Because of the complexity of MPS disorders, highly specialized, experienced, and coordinated MDTs prior to cardiac surgery are required in order to minimize negative health effects and to sustain patients' quality of life (26).

### Early Diagnosis

It has been estimated that in up to 25% of rare disease cases, delays in diagnosis ranged from 5 to 30 years (48). The general lack of diagnostic and treatment guidelines and limited access to genetic testing make early and accurate diagnosis difficult and as a result, opportunities for timely interventions can be missed. In addition, relatively common symptoms can hide underlying rare diseases, leading to misdiagnosis (49). For attenuated MPS I disorder, mean delay in diagnosis was estimated as minimum 3 years between the first physician visit and receiving a correct diagnosis (50). Cardiac signs and symptoms were some of the reasons for referral to specialist (50).

The role of clinical geneticists is important in diagnosing MPS *in utero*. Enzymatic analysis can be undertaken on fresh and cultured chorionic villi and cells from amniotic fluid (51). A prenatal molecular diagnosis is very important for fetuses at risk of MPS. It is particularly important for families with previously diagnosed MPS cases and enables pregnant females to confirm whether their fetus is likely to be affected with the same familial mutation or not. Subsequently, it helps them make a decision regarding the pregnancy.

Newborn screening has become available for MPS, but only in a few countries (52, 53) and it does not remain without flaws, such as false positives / negatives resulting in misdiagnosis (53). The limited availability and affordability of these tests remains a concern in developing countries.

### Social Support

Unmet mental healthcare needs among parents/carers of MPS patients remains an ongoing concern. Some countries have launched programmes offering an MDT approach to those patients whose diagnosis has never been confirmed (54). If mental health needs are not addressed, families may experience negative consequences additional to the already high burden and cost of their family members condition (55). Patients often seek support from personal contacts, family members and self-help groups, rather than medical professionals (56).

Awareness and knowledge of rare diseases is often lacking. As a result, many patients struggle to find adequate information. Better information provision and education on their disease, as well as greater active involvement in the decision-making of their care, may be pivotal to improve patient and family adherence to and satisfaction with regular clinic reviews (57, 58). To this end, the role of patient associations is important to promote such measures.

MPS patients need to have easy access to urgent healthcare with direct emergency access and written care plans on the electronic patient record and on the MPS passport (pendrive).

In the advanced stages of MPS, the inexorable progression of the disease makes day-to-day care a burden on the family and caregiver. Regular anticipatory review and palliative care considerations are required to provide appropriate medical care and palliative care. Finally, close relationship with their General Practitioner offers them extra support.

## Conclusions

The complexity of adult MPS disorders requires a holistic and an MDT approach to ensure the best possible outcome for the patients and their families. Cardiopulmonary complications remain the main cause of mortality in MPS disorders, resulting in increased anxiety among families and clinicians.

Since the MPS disorders are rare and there is a limited pool of metabolic specialists, international collaboration is necessary to implement full-scale training, and to increase an interest among clinicians which will subsequently impact the recruitment. Gaps in knowledge and training cannot be addressed without a formal metabolic curriculum and opportunities for fellowship in specialized centres. Given that there are very few existing tertiary centers with expertise in cardiac MPS around the world, collaborative cardiovascular research is necessary to evaluate available modalities and develop diagnostic and monitoring recommendations (guidelines) for the long-term surveillance, peri-operative cardiac intervention, and treatment outcomes. Similar initiatives for creation and dissemination of standardized transition programmes and educational tools are needed. The role of patient organizations in driving relevant national and international policies is essential.

## Author Contributions

KS and EB: conceptualization, validation, resources, and writing (review and editing). EB: guarantor. All authors contributed to the article and approved the submitted version.

## Conflict of Interest

The authors declare that the research was conducted in the absence of any commercial or financial relationships that could be construed as a potential conflict of interest.

## Publisher's Note

All claims expressed in this article are solely those of the authors and do not necessarily represent those of their affiliated organizations, or those of the publisher, the editors and the reviewers. Any product that may be evaluated in this article, or claim that may be made by its manufacturer, is not guaranteed or endorsed by the publisher.
